# Characterising Occupational Solar UVA Exposure Intensity and Self-Reported Health Outcomes Among Outdoor Military Workers in Lohatla, South Africa

**DOI:** 10.3390/ijerph23060715

**Published:** 2026-05-27

**Authors:** Sipho David Galawe, Phoka Caiphus Rathebe, Kgomotso Lebelo

**Affiliations:** 1 Department of Life Sciences, Central University of Technology Free State, Bloemfontein 9301, South Africa; sgalawe82@gmail.com; 2 Department of Environmental Health, Faculty of Health Sciences, University of Johannesburg, Auckland Park, Johannesburg 2006, South Africa; 3 Occupational and Environmental Exposure and Health Division, School of Public Health, Faculty of Health Sciences, University of the Witwatersrand, Johannesburg 2050, South Africa

**Keywords:** military personnel, ultraviolet exposure, outdoor activities, sun protection, symptoms, dermatological symptoms

## Abstract

**Highlights:**

**Public health relevance—How does this work relate to a public health issue?**

- This study addresses a well-established occupational hazard globally; however, there remains a lack of context-specific exposure data for military populations in high-radiation environments such as South Africa. The focus on military personnel undertaking physically demanding training in high-solar-radiation regions of South Africa highlights a vulnerable occupational group exposed to prolonged outdoor environmental stressors.
- By characterising field-measured solar UVA irradiance patterns and examining associated self-reported health symptoms, the study contributes evidence needed to better understand occupational solar exposure dynamics in high-intensity training environments.

**Public health significance—Why is this work of significance to public health?**

- The study draws attention to an occupational exposure affecting a sizeable and operationally critical workforce engaged in sustained outdoor activities.
- The findings provide important insights for strengthening occupational health surveillance and guiding preventive strategies aimed at reducing solar ultraviolet exposure and mitigating short-term dermatological and ocular symptoms among outdoor personnel.

**Public health implications—What are the key implications or messages for practitioners, policy makers, and/or researchers in public health?**

- The findings of this study highlight the need for public health practitioners, policymakers, and military leadership to institutionalise comprehensive sun safety programs that mandate the routine use of high-SPF sunscreen, UV-protective clothing, and other evidence-based protective measures for personnel operating in high-UV environments.
- Additionally, the findings recommend legislating solar UV exposure as an occupational health risk in outdoor professions and prioritising future research evaluating the effectiveness of sun protection interventions in high-exposure environments.

**Abstract:**

This study aimed to assess the risks associated with ultraviolet radiation (UVR) exposure among military outdoor workers at Lohatla Military Base, South Africa, and to inform targeted risk reduction strategies. A quantitative, cross-sectional design was employed, using a questionnaire survey with 161 participants (81% completion rate; 58.39% male; the largest age group was 19–25 years) and five days of objective environmental monitoring. Environmental data confirmed the presence of elevated solar ultraviolet radiation conditions, with peak irradiance levels recorded between 12:00 PM and 1:00 PM, while temperature highs frequently exceeded 35 °C (peaking at 39 °C). Statistical analysis using Spearman’s rank-order correlation revealed strong positive associations among sun protection behaviours, including wearing protective clothing, hat use, sunscreen use, and avoidance of peak sun exposure hours (ρ values up to 0.764, *p* < 0.001), indicating the clustered and interdependent nature of effective sun safety practices. Furthermore, engagement in protective behaviours was significantly associated with improved health outcomes, including a lower incidence of sunburn (ρ = 0.407, *p* < 0.001) and reduced hyperpigmentation (ρ = 0.438, *p* < 0.001). These findings indicate that combined protective strategies are associated with reduced self-reported dermatological outcomes. Despite the benefits of individual behaviours, military personnel remain exposed to high levels of environmental ultraviolet radiation, underscoring the need for institutional, evidence-based policy interventions to mitigate occupational exposure risks. The study concludes that military organisations should implement mandatory administrative controls (e.g., schedule adjustments), standardise high-ultraviolet-protection-factor protective gear, and enhance targeted health literacy training to mitigate long-term UV-related health risks and improve the operational effectiveness of their workers.

## 1. Introduction

Sunlight exposure is essential for vitamin D synthesis, which plays a crucial role in bone health and overall physiological function [1,2,3]. However, while vitamin D deficiency is linked to conditions such as osteoporosis and increased susceptibility to chronic diseases [4], excessive or uncontrolled sun exposure presents significant health risks due to ultraviolet (UV) radiation. UV radiation is a well-documented environmental carcinogen, with prolonged exposure leading to adverse health outcomes, including skin cancer, premature ageing, and ocular damage [5,6,7,8]. These risks are particularly pronounced among outdoor workers, who spend extended periods in direct sunlight in occupational settings such as construction sites, agricultural fields, and military training environments [8,9,10,11]. The UV Index, a key metric for assessing UV radiation intensity, is essential for evaluating and mitigating the risks associated with unprotected sun exposure [12]. UV radiation is known to cause DNA (Deoxyribonucleic acid) damage, which increases the risk of melanoma and non-melanoma skin cancers [6,13,14,15]. Additionally, it can lead to immune suppression, photodermatoses, and chronic skin conditions that significantly impact quality of life [16,17].

Prolonged UV exposure is a growing public health concern, particularly for outdoor workers, including construction labourers, farmers, and military personnel [6,18]. Soldiers, in particular, often endure harsh environmental conditions that require prolonged exposure to the sun during training and operational activities [9]. The combination of intense outdoor activity and variable living conditions increases their risk of UV-related health complications [19]. While the UVA irradiance serves as a proxy and crucial tool for assessing exposure risk, solar elevation and daily variations influenced by geographic and meteorological factors make it challenging to quantify and mitigate these risks effectively [12,20].

Solar ultraviolet radiation (UVR) is a recognised occupational hazard for outdoor workers, with prolonged exposure associated with dermatological and ocular health effects [21]. Although erythema is primarily driven by UVB, UVA radiation (320–400 nm) constitutes a major proportion of solar UV reaching the Earth’s surface and penetrates deeply into the dermis, contributing to oxidative stress, dermal matrix degradation, and photoaging [22,23]. Experimental studies further indicate that biological responses to UVA depend on irradiance intensity, with higher irradiance levels accelerating collagen degradation and matrix metalloproteinase activity [24]. Because UVA irradiance accounts for a large portion of terrestrial solar radiation and differs from erythemally weighted UV metrics used in UV Index calculations, direct measurement of spectral irradiance is important for accurate exposure assessment [25,26,27]. Portable electronic dosimeters are widely used for field-based UV monitoring and provide reliable measurements of environmental ultraviolet exposure [28].

Despite available data, there remains a significant gap in understanding the dynamic relationships among UV exposure, environmental conditions, and their impacts on health outcomes [29]. Many outdoor workers, including military personnel, lack awareness of appropriate sun protection measures such as wearing protective clothing, applying sunscreen, and adjusting schedules to minimise exposure during peak hours [30,31]. However, it is vital to acknowledge that personnel in structured environments such as the military often have limited autonomy to choose their attire or modify their operational schedules due to rigid institutional rules. This lack of agency, combined with the knowledge gap, not only jeopardises their health but also contributes to rising cases of skin cancer, premature ageing, and ocular damage [13]. Addressing these gaps requires targeted interventions, including awareness campaigns, improved sun protection policies implemented at the command level, and preventive strategies. Given the high-risk nature of military service, personnel require tailored interventions to mitigate UV radiation exposure effectively [10].

This study aimed to characterise field-measured solar UVA irradiance patterns during outdoor military training activities and to examine their association with self-reported dermatological and ocular health symptoms among outdoor military workers at one of South Africa’s largest military training bases. By combining objective environmental monitoring with a self-administered questionnaire, the study generated data to inform targeted strategies, such as mandatory use of high ultraviolet protection factor (UPF) gear and the implementation of administrative controls, to reduce health risks and enhance sun safety among military personnel. In addition, the conclusion demonstrates how some UV protection strategies can be applied to military operations.

## 2. Materials and Methods

**Figure 1:**
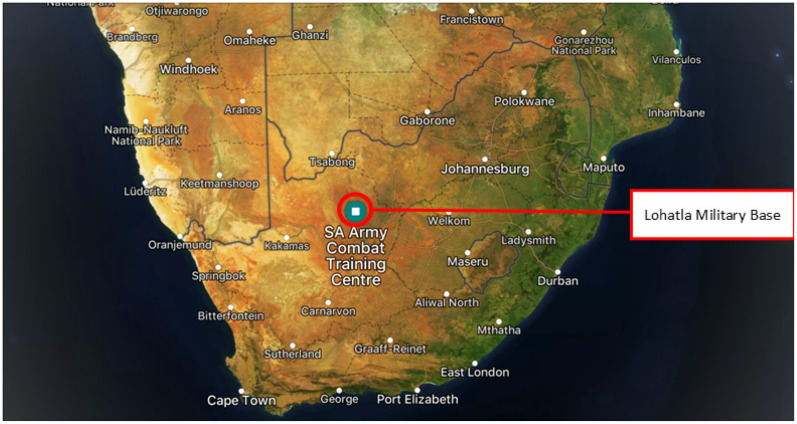
Location of the Lohatla Military Base [35].

The study was conducted at Lohatla Military Base in Kathu, South Africa (Figure 1), in the Northern Cape Province. This region is characterised by a semi-arid climate, with extremely hot summers and mild winters. According to the South African Weather Service, winter temperatures can drop to approximately 0 °C (overnight minimum), while summer temperatures frequently exceed 35 °C (daytime maximum). The area receives an average annual rainfall of 300–500 mm, primarily during the summer months. These climatic conditions result in a high frequency of clear-sky days and prolonged periods of intense sunshine, significantly increasing the likelihood of UVR (ultraviolet radiation) overexposure and associated health risks [31].

Additionally, strong seasonal winds, particularly in spring, can generate dust storms that carry urban particulate matter containing heavy metals, such as manganese. These aerosols affect the regional radiative balance by absorbing and scattering solar radiation, thereby influencing the amount of UVR reaching the Earth’s surface [32,33]. Co-exposure to both UVR and particulate matter results in a synergistic effect, intensifying oxidative stress and DNA damage in human dermal cells and potentially increasing skin irritation and accelerating skin ageing [34]. Due to manganese mining in the Northern Cape, there is a pronounced emphasis on implementing protective measures. Lohatla is strategically advantageous for military training, offering vast open spaces and diverse terrain suitable for large-scale exercises, including live fire drills and armoured-vehicle manoeuvres. Situated in the northern Kalahari Desert, the base experiences frequent periods of intense sunshine throughout the year. Its geographic location at 27°S latitude and an elevation of 1238 m above sea level contribute to high levels of solar UVR exposure, particularly for personnel engaged in outdoor activities.

### 2.1. Study Design

This study employed a quantitative, descriptive cross-sectional research design to assess the risks associated with ultraviolet radiation (UVR) exposure among military outdoor workers at Lohatla Military Base in Kathu, Northern Cape, South Africa. This methodology integrated two primary data collection streams: a structured, self-administered questionnaire to gather data on participant demographics, behaviours, and self-reported health symptoms; and objective environmental measurements of ambient UVR (Specifically broadband UVA irradiance) and heat stress Wet Bulb Globe Temperature index (WBGT) using calibrated electronic metres at representative outdoor locations across the base. The cross-sectional approach enabled the evaluation of variables at a specific point in time, facilitating immediate comparisons across different demographic groups, such as gender and ethnicity. This design provided a snapshot of the current state of UVR exposure within this population.

### 2.2. Data Collection

Data collection was conducted through a mixed-methods approach that integrated objective environmental monitoring with self-reported health outcomes. To establish a direct correlation between daily hazards and participant health, environmental data were synchronised with end-of-shift assessments on each observation day. Objective environmental data for UV (ultraviolet) radiation and heat stress were collected concurrently at each site. UV exposure was monitored throughout the peak solar gap (from 9:00 a.m. to 3:00 p.m.) using PCE-UV 40A radiometers (PCE Instruments, Meschede, Germany). At the same time, a QuesTemp 34 device (Quest Technologies, Oconomowoc, WI, USA) measured the WBGT (Wet Bulb Globe Temperature) index and its components (air temperature, humidity, air velocity, and radiant heat exposure). To evaluate the biological impact of these environmental factors, participants completed a structured, self-administered questionnaire [36] at the end of each work shift. This sequential alignment allowed for the assessment of acute health symptoms (e.g., skin erythema and ocular discomfort) in direct relation to the day’s measured peak exposure. This combined approach ensured a comprehensive understanding of the relationship between daily UV exposure and its potential health effects among military outdoor workers.

### 2.3. Solar UVR Measurement

Solar ultraviolet radiation was monitored using a PCE-UV40A broadband radiometer (PCE Instruments, Germany). The instrument measures UVA irradiance within an approximate spectral sensitivity range of 320–395 nm, with peak response centred near 365 nm, and reports irradiance in units of W m^−2^. This spectral sensitivity is further noted [29], who stated that UVR is commonly quantified in terms of spectral band irradiance, with UVA (315–400 nm) representing a major component of terrestrial ultraviolet radiation reaching the Earth’s surface. The device used in this study is designed for broadband detection of UVA radiation and therefore does not measure UVB radiation (280–315 nm). The radiometer used in this study measures only broadband UVA irradiance and does not capture UVB wavelengths; it cannot directly quantify erythemally weighted UV or UVI. Moreover, it is assumed that the instrument will likely differ in peak UVA irradiance values depending on the measurement tool, likely due to differences in instrumentation, spectral response, calibration, and measurement design. Therefore, the data are not absolute atmospheric benchmarks; the reported irradiance values should be interpreted as portable field measurements of UVA intensity within an occupational context.

Accordingly, measurements obtained in this study are reported as UVA irradiance, representing the physical radiant flux density within the UVA spectral band. While UVA contributes less to erythemal response compared with UVB, variations in UVA irradiance still reflect changes in overall solar ultraviolet intensity associated with solar elevation and atmospheric conditions. In this study, the instrument was deployed under field conditions to characterise temporal patterns of solar UVA irradiance experienced during outdoor military training activities. The equipment was factory-calibrated against traceable radiometric standards and field-verified under local sunlight conditions. A pre-deployment calibration check validated the sensor’s wavelength response and irradiance measurements against a reference UV source, complemented by daily checks with a secondary calibrated metre to ensure reliability. In deployment, devices were mounted in flat, unobstructed positions approximately 1.5 m above ground on stable stands at four fixed, representative field locations to capture relevant outdoor radiation levels across military activities: static access control, lawn maintenance duties (e.g., operating lawn mowers), pre-deployment training, and high-intensity training (e.g., physical training drills and musketry). Ambient readings recorded near each specific task setting were used to estimate exposure for that activity. Data collection covered the whole day, with devices retrieved at shift-end for data download and a brief calibration assessment. Ambient UVA irradiance measurements were systematically conducted on five carefully selected working days across four months: 1 December 2023; 1 January 2024; 1 February 2024; 1 March 2024; and 29 March 2024. This design enhanced time-based spacing to enable reliable cross-month exposure comparisons and to capture normal environmental fluctuations throughout the monitoring period. All UVA irradiance monitoring data points were time-synchronised, centrally stored, and carefully marked with necessary metadata, including the date, location, device identification number, current weather, and relevant operating notes.

### 2.4. Heat Stress Exposure Measurement

Heat stress was assessed at multiple outdoor work locations, strategically selected to reflect varying job tasks and potential exposure profiles across different workstations. At each representative site, a QUEST Technologies QuesTemp 34 device was mounted on a tripod at a standardised height of approximately 1.1 m above the ground to minimise obstruction and ensure consistent readings. The WBGT (Wet Bulb Globe Temperature) index, an integrated measure utilised to evaluate occupational heat stress, incorporates air temperature (dry bulb), humidity (wet bulb), air velocity, and radiant heat exposure (globe temperature) [37]. Measurements were systematically recorded during the typical peak heat exposure period, from 9:00 a.m. to 3:00 p.m., with each location undergoing a 15 min stabilisation period before the formal data collection commenced. The device’s reliability was verified through rigorous calibration procedures conducted at the start and end of every measurement day, confirming accuracy levels of ±0.5 °C for dry bulb temperatures (0–120 °C) and ±5% RH (relative humidity) (20–95% RH). These checks involved validating sensor responses against reference standards and confirming globe temperature via a controlled warm-up procedure, ensuring a representative assessment of heat stress risk across the diverse study area tasks.

### 2.5. Questionnaires

The study recognised that health-related symptoms among outdoor workers are multifactorial, not solely attributable to UV exposure. To address multiple potential causes, a mixed-methods approach combined objective UVR measurements with a self-administered 22-item questionnaire (Supplementary Material S1). This instrument was structured into three sections: Demographic Information (age, gender, ethnicity, education), Work-Related Factors (job titles, outdoor exposure duration, sunscreen and protective gear use, UV risk awareness), and Health-Related Symptoms, which included both acute presentations (excessive tearing, dry eyes, blurred vision, sunburn, sunspots, and dry skin) and chronic indicators (bumps, brown spots/hyperpigmentation, wrinkles). To facilitate precise correlation between daily exposure and immediate health effects, participants completed the questionnaire at the end of each work shift on every day that ambient UV radiation measurements were taken. To further outline UV-driven effects in the context of other environmental stressors, contextual covariates such as wind and dust, mentioned in the study’s introduction, were recorded and incorporated into the analysis. The study acknowledged that long-term skin changes, such as wrinkles, are complex outcomes resulting from cumulative factors, including ageing, genetics, and chronic UV exposure. The questionnaire was completed by all consenting participants, enabling a comprehensive integration of objective UVR data with self-reported health outcomes to provide a holistic assessment of occupational health risks.

### 2.6. Participants and Recruitment

Participants were recruited through the Lohatla Military Base training officer and fully informed about the research objectives, the radiometers used to measure UVR exposure, and the study duration. They were notified of the right to withdraw at any time without affecting their military status or training opportunities. The sample covered all four arms of service, including the Army, Air Force, Navy, and the South African Military Health Service (SAMHS), to assess differences in UVR exposure by occupational role and branch-specific environmental conditions. Participants were selected based on specific inclusion criteria to ensure a relevant and representative sample. The study focused on adults (18 years or older) serving in the South African National Defence Force (SANDF) and engaged in outdoor military operations, ensuring uniform exposure to UVR. To minimise potential biases, individuals with a family history of cataracts or skin cancer were excluded to reduce genetic predisposition effects. Additionally, participants with prior outdoor work experience were excluded to mitigate the influence of previous UVR exposure on the study’s findings.

### 2.7. Sampling Procedure and Size

The study population comprised 419 military personnel engaged in outdoor activities at the Lohatla Military Base. A stratified systematic sampling method was utilised to ensure proportional representation across different experience levels. Participants were organised into four strata based on their years of service in the SANDF: ≤2 years, >2 but ≤5 years, >5 but ≤10 years, and >10 years. Within each stratum, potential volunteers were selected by choosing every second individual from the experience category lists. A target sample size of 200 was determined using the Centers for Disease Control and Prevention (CDC) EPINFO software (version 7.2.2.6) for cross-sectional studies. The sample size calculation assumed a conservative prevalence estimate to maximise statistical power, given the absence of prior exposure data specific to this population. It yielded a minimum required sample size of 200 participants, based on a 5% margin of error at a 95% confidence level, with adjustment for covariates [38,39]. Of the target sample, 161 participants provided informed consent and completed the study, resulting in a participation rate of approximately 81%, which is considered acceptable for this research study. However, it is important to note that *n* = 161 is lower than the targeted 200 participants, resulting in a ±8% margin of error. Therefore, the reduced participation rate widened the confidence intervals, and findings should therefore be interpreted as indicative rather than population-precise estimates.

### 2.8. Validity and Reliability of Data Collection Tools

The variables of interest included knowledge of the effects of UV exposure on skin and health, attitudes towards sun protection, and sun protection practices. After reviewing existing literature on public knowledge of sun protection across various countries, a questionnaire based on validated items was developed, thereby improving the research’s validity [40]. To evaluate the validity and reliability of the assessment tools for the primary study and to refine the questions for improved comprehension, a pilot study was conducted with 20 SANDF members. These subjects were excluded from the study to prevent bias. Self-administered questionnaires were used to collect data, and participants completed questionnaires after obtaining consent. Additionally, an electronic UVR radiometer was used to monitor ambient ultraviolet radiation levels, and the Wet Bulb Globe Temperature (WBGT) index was used to monitor heat stress during training exercises, providing essential insights into how temperature and humidity can influence UV exposure. This comprehensive data collection approach enhanced the robustness of the findings and provided a holistic understanding of the interplay between military training, UV radiation risks, and heat stress. The study was conducted between December 2023 and March 2024 to allow for thorough data collection and to ensure a wide range of responses, thereby increasing the study’s reliability. The questionnaire design was based on previous well-established research, ensuring its accuracy and relevance [40].

### 2.9. Ethical Clearance

Before the commencement of the study, the researcher obtained ethical approval from the relevant ethics committees, such as the 1 Military Hospital Research Ethics Committee (1MHREC; Approval Number: 1MH/302/6/02.10.2023) and the Faculty of Health Sciences Research Ethics Committee at the University of the Free State (UFS HSREC; Approval Number: UFS-HSD2023/1227/2811). Furthermore, consent was acquired from all study participants. All phases of the study were conducted in compliance with the Declaration of Helsinki and the Protection of Personal Information Act.

### 2.10. Data Analysis

Statistical analysis was conducted using the Statistical Package for the Social Sciences (SPSS), specifically IBM SPSS Statistics for Windows, version 26.0 (Armonk, NY, USA: IBM Corp). Descriptive statistics, including frequencies (*n*), percentages (%), and means ($\bar{x}$) and standard deviations (SD), were used to summarise the socio-demographic characteristics of the study participants and the environmental UV measurements. The normality of continuous variables was assessed using the Shapiro–Wilk test [41]. Inferential statistical analysis was conducted to examine relationships between sun protection behaviours, exposure conditions, and self-reported health outcomes. Given that most variables were measured using ordinal Likert-scale responses and binary categories, Spearman’s rank-order correlation coefficient (ρ) was used as the primary measure of association. Continuous variables were assessed for normality using the Shapiro–Wilk test. Where appropriate, Pearson’s correlation coefficient (r) was calculated for normally distributed continuous variables as a sensitivity analysis to confirm the robustness of observed relationships. All statistical tests were two-tailed, and a *p*-value of <0.05 was considered statistically significant.

## 3. Results

Initially, 200 participants were invited to the study, and upon providing consent and completing the questionnaire, the completion rate was 81%, with 161 respondents. The questionnaire survey was completed by 161 military outdoor workers, comprising males (*n* = 94, 58.39%) and females (*n* = 67, 41.62). The age category with the largest study population was 19 to 25 years (*n* = 51, 31.68%). Most study participants held a matric certificate (Completed high school education) (*n* = 115, 71.43%) as the highest qualification, with the least being postgraduate (*n* = 2, 1.24%). Table 1 outlines the socio-demographic characteristics of study participants.

Most participants had employment durations of one month to two years, with the fewest having employment durations of four to six years. The highest age category had the fewest participants (41 years or older), while the 19 to 25-year-old age category had the most. In addition, males accounted for the highest proportion of study participants.

Table 2 summarises relationships between sun protection behaviours and health outcomes, providing insights into the protective behaviours of military outdoor workers and their associated health outcomes.

Table 2 presents the results of the Spearman correlation analysis examining relationships between sun protection behaviours, exposure conditions, and self-reported health outcomes. The correlation analysis revealed consistent clustering of protective behaviours, with strong positive associations among clothing use, sunscreen importance, and avoidance of peak sun exposure. These findings indicate that sun protection behaviours are not adopted in isolation but rather as part of an integrated behavioural pattern. Strong positive correlations were observed among protective behaviours, indicating behavioural clustering. Wearing clothes that cover the arms and legs was strongly associated with avoiding sun exposure between 12:00 and 15:00 (ρ = 0.659, *p* < 0.001) and with the importance of sunscreen (ρ = 0.619, *p* < 0.001). Similarly, the use of a hat showed a very strong positive correlation with wearing protective clothing (ρ = 0.764, *p* < 0.001) and a strong correlation with avoiding peak sun exposure (ρ = 0.673, *p* < 0.001). The importance of sunscreen was also strongly associated with avoiding peak sun exposure (ρ = 0.612, *p* < 0.001).

With respect to exposure conditions, working around reflective surfaces showed no statistically significant association with blurred vision (ρ = 0.017, *p* = 0.830) and a weak, non-significant association with the importance of sunscreen (ρ = 0.152, *p* = 0.055). Associations between protective behaviours and health outcomes demonstrated consistent patterns. Sunscreen application was moderately associated with reduced sunburn (ρ = 0.407, *p* < 0.001) and reduced hyperpigmentation (ρ = 0.438, *p* < 0.001). Covering the arms and legs was also moderately associated with reduced sunburn (ρ = 0.435, *p* < 0.001). Avoiding peak sun exposure was associated with reduced hyperpigmentation, with a weak-to-moderate association (ρ = 0.244, *p* = 0.002). In addition, a weak but statistically significant association was observed between sunscreen use and reduced eye pain (ρ = 0.218, *p* = 0.006).

In this study, “brown spots” refer to hyperpigmented lesions as indicators of cumulative ultraviolet exposure. While solar lentigines are more commonly observed in lighter skin types, post-inflammatory hyperpigmentation and uneven skin tone are more prevalent in darker skin types (Fitzpatrick IV–VI), which comprised the majority of the study population.

### Environmental UV Measurements and UVA Irradiance

Measurements were taken to assess and compare the outdoor temperature over the five days. The readings were taken at seven intervals per day to account for the changing incident angle of the sun between 09:00 a.m. and 3:00 p.m., as shown in Table 3:

Table 4 presents descriptive statistics for field-measured solar UVA irradiance patterns recorded at different times of day during the training period. The results demonstrate clear temporal variation in irradiance levels, with lower values observed during early-morning periods and progressively higher values recorded as solar elevation increased toward midday. Peak irradiance values were consistently observed during the midday interval, i.e., 12 W·m^−2^ on days two and three, between 12:00 and 1:00 p.m. The lowest UVA irradiance was recorded on days three and four (09:00 a.m.). This pattern reflects expected solar radiation dynamics driven by changes in solar elevation and atmospheric path length. Minor day-to-day variability was observed, likely attributable to atmospheric conditions such as cloud cover and aerosol presence, but overall temporal trends remained consistent across measurement days. These findings highlight distinct periods of elevated ultraviolet exposure during the training day, particularly during midday, when outdoor personnel are likely to experience the greatest solar radiation intensity.

## 4. Discussion

This study characterised the temporal patterns of field-measured solar UVA irradiance during outdoor military training. It examined its relationship with self-reported dermatological and ocular symptoms among personnel operating in a high-solar-radiation environment. Given the cross-sectional design and reliance on self-reported symptoms, causal relationships between UVA exposure and health outcomes cannot be established.

The findings demonstrate clear diurnal variability in UVA irradiance, with peak intensities occurring during midday periods corresponding to maximum solar elevation. These exposure patterns are consistent with well-established solar radiation dynamics, whereby higher solar elevation leads to greater ultraviolet irradiance at the Earth’s surface. The concurrent measurement of WBGT highlights the combined burden of heat and solar radiation exposure, which may act synergistically to increase physiological strain and influence behavioural responses such as reduced use of protective clothing. Although the instrument used in this study measured broadband UVA irradiance rather than erythemally weighted ultraviolet radiation, the observed exposure patterns provide important insight into the intensity of occupational solar exposure experienced by military personnel engaged in prolonged outdoor activities. The results, therefore, contribute to the growing body of evidence highlighting the occupational health relevance of solar ultraviolet exposure among outdoor workers.

The study’s results provided critical insights into the demographics of outdoor military workers, highlighting a youthful cohort aged 19 to 25 years, with significant implications for training and operational strategies. The gender distribution indicated 58.39% male and 41.62% female participants, highlighting the need for inclusive practices that ensure equal opportunities. Notably, 71.43% of respondents held only a matric certificate (High school qualification), showing a gap in advanced qualifications that could affect job performance and adaptability. This highlighted the urgent need for targeted educational programmes to enhance skill sets and improve operational effectiveness. An 81% completion rate reflected strong participant engagement, reinforcing the reliability of findings that accurately represented the military outdoor workers in Lohatla. Utilising this information, military organisations could modify their training initiatives to align with their personnel’s demographics. Recognising the mental and physical demands of these younger soldiers could help develop support systems that promote resilience and well-being. The results also served as a valuable resource for leadership in making evidence-based workforce management decisions. Ultimately, these insights clarified the state of outdoor military workers and provided actionable pathways for professional development and job satisfaction.

The strong correlations observed among protective behaviours suggest an integrated behavioural response to perceived environmental risk. Rather than acting independently, sun protection measures appear to be adopted collectively, suggesting that interventions targeting one behaviour may have spillover effects on other behaviours. This has important implications for occupational health interventions, as multi-component strategies may be more effective than single-measure approaches in high-exposure environments such as military training settings. The findings indicate that protective behaviours are strongly interrelated, suggesting that sun safety practices are adopted in clusters rather than as isolated actions [13,16,17]. This behavioural clustering is consistent with previous research showing that individuals who engage in one protective behaviour are more likely to adopt additional protective strategies.

Contrary to expectations, no statistically significant association was observed between working around reflective surfaces and blurred vision. This may reflect variability in individual exposure conditions, the influence of other environmental factors, such as dust and wind, or limitations of self-reported symptom data. Protective behaviours were consistently associated with improved health outcomes. Sunscreen use, protective clothing, and avoidance of peak sun exposure were all associated with reduced reports of sunburn and hyperpigmentation. These findings reinforce the effectiveness of combined sun protection strategies in mitigating the adverse effects of solar ultraviolet exposure [19,31].

### 4.1. Socio-Demographic Factors

The demographic data from a questionnaire survey of 161 military outdoor workers reveal key characteristics in gender, age, and educational attainment, consistent with prior studies of military populations. A male predominance (58.39%) is observed in the study population, aligning with the general demographics of military roles, which traditionally include a larger male representation, particularly in outdoor and physically demanding roles [42]. This gender distribution reflects recruitment and role assignment patterns across military institutions worldwide, where physical endurance requirements and exposure to environmental elements are often associated with higher male participation [42]. The largest age category among respondents, 19 to 25 years (31.68%), highlights the youthfulness common in outdoor military roles. This demographic pattern is supported by research suggesting that younger individuals are more likely to engage in physically demanding outdoor military roles, which require significant physical resilience and adaptability to harsh environments [43]. Younger military personnel, such as those in this age bracket, are also reported to have unique occupational health considerations, including greater susceptibility to long-term physical strain and environmental exposures [44,45]. Additionally, predominantly younger workers in outdoor roles may require targeted occupational health and safety interventions to promote long-term well-being.

Educationally, most participants (71.43%) completed matric (Grade 12, the final high school qualification), with only a tiny percentage (1.24%) holding postgraduate degrees. The matric certificate represents the successful completion of High School, the final year of secondary education in South Africa. This trend aligns with typical educational levels for entry-level military roles in outdoor settings, where higher academic qualifications are not usually mandatory. Lower educational attainment within this group may impact workers’ health literacy and understanding of occupational health risks. Military personnel with higher educational backgrounds often report a greater awareness of occupational hazards, which may influence adherence to safety protocols and health outcomes over time. The data from this survey aligns closely with existing literature, highlighting demographic factors such as gender, age, and education that shape the occupational health landscape for military outdoor workers. Future interventions should consider these factors, focusing on health literacy, resilience training, and gender-sensitive support mechanisms to address the unique occupational risks faced by this demographic [45].

### 4.2. Environmental UV Measurements and UVA Irradiance

The temperature data collected over five days revealed notable extremes, with a peak temperature of 39 °C recorded on days 3 and 5 at 14:00, while the lowest temperature recorded was 26 °C on day 1 at 09:00. This variation indicates a significant range in temperature that could have implications for environmental or occupational conditions, as temperature extremes are known to affect worker productivity and health [21,46,47]. Temperature measurements showed a typical diurnal pattern, with lower values in the morning and peak temperatures in the early afternoon. Although day-to-day variation was present, overall patterns were consistent with expected environmental conditions in semi-arid climates. Peak temperatures exceeding 35–39 °C indicate substantial heat exposure during operational hours, reinforcing the combined burden of thermal and solar stress experienced by personnel. Such findings align with previous research suggesting that environmental temperature readings may show variability without significant implications for health or occupational outcomes. This insight is crucial for understanding the average environmental conditions in the studied setting. However, it simultaneously highlights the importance of continuously monitoring temperature variations and specific peak measurements for potential impacts on health and performance in similar contexts, as those extremes still pose acute risks [21,25].

Peak UVA irradiance values reached approximately 12 W·m^−2^ during midday (12:00–13:00), corresponding to the periods of maximum solar elevation on days two and three. Conversely, the lowest UVA irradiance of 3 was observed on days 3 and 4 at 09:00, suggesting a significantly reduced risk of UV-related harm during this time. This finding suggests that although peak levels were recorded, no substantial deviation from expected diurnal patterns was observed that would alter the overall exposure risk profile. The observed UVA irradiance patterns follow well-established solar radiation dynamics, characterised by a pronounced diurnal cycle driven primarily by solar elevation. Irradiance increased progressively from morning to midday, where peak values were consistently observed, before declining toward the afternoon. This temporal pattern is expected under clear-sky conditions typical of semi-arid environments such as Lohatla. Day-to-day variability in irradiance magnitude likely reflects changes in atmospheric conditions, including cloud cover and aerosol loading, rather than systematic differences in exposure conditions. These findings indicate that occupational exposure risk is primarily governed by predictable within-day temporal patterns, with midday periods representing the highest exposure window for outdoor personnel.

These results imply that, while the overall analysis did not reveal significant differences, specific days showed variations in UV exposure levels that warrant further attention, particularly for sun safety strategies [48]. During the five-day observation period, varying levels of risk from unprotected sun exposure were identified and categorised as high to extreme. Extreme risk levels were noted on day one from 11:00 to 13:00, on day two from 11:00 to 13:00, and on day three from 11:00 to 14:00. High-risk levels were present on days four and five from 11:00 to 14:00, correlating with UVA irradiance values between 6 and 10. These findings emphasise the critical need for sun protection during peak solar elevation, particularly in settings where individuals are at risk of prolonged sun exposure [22,24,49]. The consistently high UVA irradiance readings during specific periods highlight the necessity for effective sun protection strategies to mitigate health risks associated with UV exposure.

### 4.3. Limitations

This study has several limitations that should be considered when interpreting the findings. The sample size limits the precision of estimated proportions and reduces the ability to detect smaller effect sizes. Solar ultraviolet radiation was measured using a broadband radiometer designed to quantify UVA irradiance within the approximate spectral range of 320–395 nm. The instrument does not measure UVB radiation, nor does it directly quantify erythemally weighted ultraviolet radiation, which forms the basis of the Ultraviolet Index (UVI) [26,27]. Moreover, because UVA contributes less to erythemal response than UVB, the measured irradiance values may not directly reflect acute sunburn risk, which is more strongly influenced by UVB radiation. Consequently, the study reports measured UVA irradiance as an indicator of solar ultraviolet exposure intensity rather than a direct measure of erythemal risk. Ambient UVA irradiance measurements were used as a proxy for potential exposure; however, individual-level exposure may differ due to task variability, body orientation, clothing, and time–activity patterns. Additionally, health outcomes were based on self-reported symptoms, which may be subject to recall bias or individual differences in perception. Despite these limitations, the study provides valuable field-based insights into occupational solar exposure patterns and associated health symptoms among military personnel operating in high-radiation environments.

## 5. Conclusions

This study profiles military outdoor workers at a major South African training base and quantifies environmental UV exposure, providing essential evidence for institutional sun safety reform. Environmental monitoring confirmed prolonged high-irradiance conditions, with peak UVA irradiance values reaching approximately 12 W·m^−2^ during midday operational periods (11:00–14:00). These data indicate that the environment is characterised by elevated solar UV conditions that may increase exposure risk. Among a predominantly youthful workforce (ages 19–25) with entry-level education, protective behaviours were positively correlated (e.g., use of bush hats with protective clothing); however, uniform regulations and fixed schedules constrain practical implementation, highlighting that awareness alone is insufficient and that policy-level changes are necessary to enable protective measures such as rescheduling high-exposure tasks outside peak UV hours. Correlation analysis demonstrated that sun protection behaviours are strongly interrelated and collectively associated with reduced dermatological and ocular symptoms. These findings highlight the importance of integrated sun safety strategies rather than reliance on single protective measures. These findings support targeted institutional interventions, including command-level policy mandating wide-brim hats and high-UPF uniforms, standard-issue high-sun-protection-factor sunscreen, and administrative controls to reduce exposure during UV peaks. Educational programmes should be tailored to entry-level workers’ backgrounds, emphasising practical risk management and the importance of comprehensive sun protection.

## Figures and Tables

**Table 1:** Participants’ socio-demographics and occupation data.

Socio-Demographic		Frequency (*n*)	Percentage (%)
Age	19 to 25 years old	51	31.68%
	26 to 30 years old	34	21.12%
	31 to 40 years old	46	28.57%
	41 years or older	30	18.63%
Gender	Female	67	41.62%
	Male	94	58.39%
Education level	Matric	115	71.43%
	Diploma	34	21.12%
	Degree	10	6.21%
	Postgraduate degree	2	1.24%
Ethnicity	Black	137	85.09%
	White	10	6.21%
	Indian	10	6.21%
	Coloured	4	2.49%
	Other	0	0%
Occupation	Instructor	5	3.11%
	Newly appointed/MSDS	43	26.70%
	Student on course	0	0%
	Doing re-training	1	0.62%
	Preparing for deployment	41	25.47%
	Access control/Guard	32	19.88%
	Other	39	24.22%
Duration of Employment	1 month to 2 years	102	63.35%
	>2 years to ≤4 years	20	12.42%
	>4 years to ≤6 years	17	10.56%
	>6 years	22	13.67%

**Table 2:** Correlational analysis of sun protection behaviours and health outcomes.

Summary of Key Correlations					
Categories	Variable 1	Variable 2	ρ (Spearman)	*p*-Value	Interpretation
UV Exposure and Protective Measures	Wearing Clothes that Cover Arms & Legs	Avoiding Sun (12:00–3:00 p.m.)	0.659	<0.001	Strong positive correlation: Individuals who wear protective clothing are more likely to avoid peak sun exposure hours.
		Sunscreen Use	0.619	<0.001	Strong positive correlation: Those who cover up are also more likely to value sunscreen use.
	Working Around Reflective Surfaces	Sunscreen Use	0.152	0.055	Weak positive correlation (not statistically significant): Individuals exposed to reflective surfaces tend to value sunscreen slightly, but the relationship is not statistically significant.
UV Exposure and Eye Health Symptoms	Working Around Reflective Surfaces	Blurred Vision	0.017	0.830	No meaningful correlation: Exposure to reflective surfaces was not associated with blurred vision in this dataset.
Protective Behaviours	Using a Hat	Wearing Clothes that Cover Arms/Legs	0.764	<0.001	Very strong positive correlation: Hat use is strongly associated with wearing protective clothing, indicating clustered protective behaviours.
		Avoiding the Sun Between 12:00 and 3:00 p.m.	0.673	<0.001	Strong positive correlation: Individuals who wear hats are also more likely to avoid peak sun exposure.
	Sunscreen Use	Avoiding the Sun Between 12:00 and 3:00 p.m.	0.612	<0.001	Strong positive correlation: Those who value sunscreen use are more likely to avoid peak sun hours.
Health Outcomes and Protective Measures	Brown Spots/Hyperpigmentation	Sunscreen Use	0.438	<0.001	Moderate association indicating that higher reported sunscreen use corresponds with lower reporting of hyperpigmentation-related outcomes
		Avoiding the Sun Between 12:00 and 3:00 p.m.	0.244	0.002	Weak to moderate positive correlation: Avoiding peak sun exposure is associated with reduced hyperpigmentation.
	Sunburn	Sunscreen Use	0.407	<0.001	Moderate positive correlation: Sunscreen use is associated with a lower incidence of sunburn (protective effect).
		Covering Arms and Legs	0.435	<0.001	Moderate positive correlation: Wearing protective clothing is associated with a reduced risk of sunburn.
	Eye Pain	Sunscreen Use	0.218	0.006	Weak positive correlation: Sunscreen use shows a slight association with reduced eye pain symptoms.

Interpretation of correlation strength: weak (ρ = 0.10–0.29), moderate (ρ = 0.30–0.49), strong (ρ ≥ 0.50). Positive correlations indicate that higher levels of protective behaviour are associated with reduced adverse health outcomes.

**Table 3:** Outdoor temperature during the measurement period.

Days	Time of the Day	Outdoor Temperature (°C)	Min.	Max.	Range	Mean ± SD
1	09:00	26	26	36	10	31.86 ± 3.761
1	10:00	28				
1	11:00	32				
1	12:00	32				
1	13:00	33				
1	14:00	36				
1	15:00	36				
2	09:00	28	28	37	9	33.57 ± 3.460
2	10:00	30				
2	11:00	33				
2	12:00	35				
2	13:00	37				
2	14:00	37				
2	15:00	35				
3	09:00	32	32	39	7	36.14 ± 2.410
3	10:00	34				
3	11:00	36				
3	12:00	37				
3	13:00	37				
3	14:00	39				
3	15:00	38				
4	09:00	29	29	38	9	34.43 ± 3.259
4	10:00	32				
4	11:00	34				
4	12:00	34				
4	13:00	38				
4	14:00	38				
4	15:00	36				
5	09:00	30	30	39	9	34.71 ± 3.147
5	10:00	32				
5	11:00	34				
5	12:00	35				
5	13:00	38				
5	14:00	39				
5	15:00	35				

**Table 4:** Field-measured solar UVA irradiance intensity patterns by time of day.

Days	Time of the Day	UVA Irradiance (W·m^−2^)	Min.	Max.	Range	Mean ± SD
1	09:00	4	4	11	7	9 ± 2.582
1	10:00	8				
1	11:00	11				
1	12:00	11				
1	13:00	11				
1	14:00	10				
1	15:00	8				
2	09:00	4	4	12	8	9 ± 3.055
2	10:00	7				
2	11:00	11				
2	12:00	12				
2	13:00	12				
2	14:00	10				
2	15:00	7				
3	09:00	3	3	12	9	9 ± 3.416
3	10:00	7				
3	11:00	11				
3	12:00	12				
3	13:00	12				
3	14:00	11				
3	15:00	7				
4	09:00	3	3	10	7	7.29 ± 2.628
4	10:00	7				
4	11:00	10				
4	12:00	10				
4	13:00	9				
4	14:00	7				
4	15:00	5				
5	09:00	4	4	10	6	6.57 ± 2.070
5	10:00	5				
5	11:00	8				
5	12:00	10				
5	13:00	7				
5	14:00	7				
5	15:00	5				

## Data Availability

The data that support the findings of this study are available from the authors upon reasonable request.

## Supplementary Materials

The following supporting information can be downloaded at: https://www.mdpi.com/article/10.3390/ijerph23060715/s1, File S1: Questionnaire.
