# Supplementary material for: Characterising Occupational Solar UVA Exposure Intensity and Self-Reported Health Outcomes Among Outdoor Military Workers in Lohatla, South Africa
Source: Int J Environ Res Public Health. 2026 May 27;23(6):715. doi: 10.3390/ijerph23060715 (PMC13299541; doi:10.3390/ijerph23060715)
Supplement: Supplementary file 1 [file ijerph-23-00715-s001.zip › ijerph-4229851-supplementary.pdf]

Questionnaire	

Participant Study Code:

Assessment of ultraviolet radiation exposure among military outdoor workers in Lohatla, Northern Cape.

DATE: ……………………

1. This study adapted the questionnaire from the studies of Ultraviolet radiation exposure, risk, and protection in military and outdoor athletes and Pilot awareness and preventative measures of Ultraviolet radiation effects at altitude. This instrument was developed by Janjani et al. (2019). The purpose of this study is to assess health effects as well as environmental and personal exposure to ultraviolet radiation. The specific articles were chosen because of the similarity in methodology.

2. You are invited to participate in a research project: **Assessment of ultraviolet radiation exposure among military outdoor workers in Lohatla, Northern Cape.** Participation in this study is voluntary, and an individual can withdraw at any time with no negative consequences for themselves. The questionnaire is about the assessment of the health effects and personal exposure to ultraviolet radiation when you are performing your daily duties.

SECTION A: DEMOGRAPHIC INFORMATION (*Please mark with an "X")*

1. What is your age………..years?

2. What is your gender?

☐ Male

☐ Female

☐ Prefer not to say

3. What is your ethnicity?

☐ Black/African

☐ White/Caucasian

☐ Indian

☐ Coloured

☐ Other

If other, please specify:_______________________________________

4. What is your highest level of education?

☐ Matric/Grade 12 ☐ Diploma ☐ Bachelor's ☐ Postgraduate degree

SECTION B: WORK-RELATED QUESTIONS

5. What is your current job title? (**Tick the applicable job title**)

☐ Instructor

☐ Newly appointed/MSDS

☐ Student on course

☐ Doing re-training

☐ Preparing for deployment

☐ Access control/Guard

☐ Other

If other, please specify____

6. How long have you been working (as indicated above) …………years …….... months? And what is your primary work location? ____

7. When working outdoors during the day, how long are you exposed to the sun? (**Time in hours**)

☐ (0-3)

☐ (4-7)

☐ (5-8)

☐ (More than 8)

8. Before the start of the work shift, do you apply sunscreen on all exposed areas?

☐ Never ☐ Rarely ☐ Sometimes ☐ Always

8.1. If yes, when do you normally put it on?

☐ Before I go outside (before I leave my home)

☐ As soon as I am in the sun

☐ After being in the sun for a while, but before I realize that my skin has turned red

☐ If I realize that my skin has turned red

And what is the UVA/UVB protection level of the sunscreen applied, please tick below?

SPF 15 ☐ SPF 30 ☐ SPF 50 ☐ SPF 100 ☐

9. Protective clothing (select all that apply). If you are working outside in the sun, it is important to wear…

☐ Bush Hat

☐ Sunglasses/Goggles

☐ Cap

☐ Long-sleeve shirt

☐ Other

If other please specify____.

10. How much do you agree with the following statements?

If you are working outside in the sun, it is important to wear…

	**Disagree a lot**	**Disagree**	**Neither disagree or agree**	**Agree**	**Agree a lot**
Use a hat	(1) ❑	(2) ❑	(3) ❑	(4) ❑	(5) ❑
Wear clothes that cover your arms and legs	(1) ❑	(2) ❑	(3) ❑	(4) ❑	(5) ❑
Avoid the sun between 12 and 3 pm	(1) ❑	(2) ❑	(3) ❑	(4) ❑	(5) ❑
Use sunscreen	(1) ❑	(2) ❑	(3) ❑	(4) ❑	(5) ❑

11. Do you work around reflective surfaces?

☐ Yes, concrete

☐ Yes, grass

☐ No

SECTION C: HEALTH EFFECTS OF UVR EXPOSURE

The following questions are based on the previous 12 months (*Please mark with an "X")*.

12. Have you noticed any signs of sunburn? ☐ Yes ☐ No

13. Have you experienced any of the following signs & symptoms related to your eyes during/after outdoor tasks? (***Please mark with an "X" all that exists).***

☐ Excess tearing

☐ Dry eyes

☐ Blurred vision

☐ Eye pain

☐ Eye redness

☐ Light sensitivity

☐ Lazy eye

☐ Night blindness

☐ Eye strain

14. Have you experienced any of the following signs and symptoms appearing on your body after outdoor tasks? (***Please mark with an "X" all that exists).***

	**Disagree a lot**	**Disagree**	**Neither disagree or agree**	**Agree**	**Agree a lot**
Soft bump from sunburn after outdoor activities	(1) ❑	(2) ❑	(3) ❑	(4) ❑	(5) ❑
Brown spots	(1) ❑	(2) ❑	(3) ❑	(4) ❑	(5) ❑
Tender, red bump	(1) ❑	(2) ❑	(3) ❑	(4) ❑	(5) ❑
A large brownish spot with darker sparkles	(1) ❑	(2) ❑	(3) ❑	(4) ❑	(5) ❑
A painful lesion that is itchy or burns	(1) ❑	(2) ❑	(3) ❑	(4) ❑	(5) ❑

15. Have you experienced any of the following signs and symptoms related to your skin during/after outdoor tasks? (***Please mark with an "X" all that exist).***

	**Disagree a lot**	**Disagree**	**Neither disagree or agree**	**Agree**	**Agree a lot**
Dry or itchy skin on exposed areas	(1) ❑	(2) ❑	(3) ❑	(4) ❑	(5) ❑
Wrinkles or fine lines on sun-exposed areas	(1) ❑	(2) ❑	(3) ❑	(4) ❑	(5) ❑
Sunburn in the past 12 months	(1) ❑	(2) ❑	(3) ❑	(4) ❑	(5) ❑
Skeletal/bony hands	(1) ❑	(2) ❑	(3) ❑	(4) ❑	(5) ❑
Sunspots or brown patches on sun-exposed areas	(1) ❑	(2) ❑	(3) ❑	(4) ❑	(5) ❑

16. How much do you agree with the following statements (awareness)? (***Please mark with an "X" all that exists.)*** You are exposed to ultraviolet radiation …

	**Disagree a lot**	**Disagree**	**Neither disagree or agree**	**Agree**	**Agree a lot**
When you are in the shade	(1) ❑	(2) ❑	(3) ❑	(4) ❑	(5) ❑
When you are in the sun without sunbathing	(1) ❑	(2) ❑	(3) ❑	(4) ❑	(5) ❑
When you are in the water	(1) ❑	(2) ❑	(3) ❑	(4) ❑	(5) ❑
When it is cloudy	(1) ❑	(2) ❑	(3) ❑	(4) ❑	(5) ❑
When it rains	(1) ❑	(2) ❑	(3) ❑	(4) ❑	(5) ❑

17. Any other comments related to health effects due to exposure to ultraviolet radiation:

……………………………………………………………………………………………………
……………………………………………………………………………………………………
…………………………………………………………………………………………………….

Thank you for your time; it is much appreciated.
